# Quantum Dot–Polyfluorene Composites for White-Light-Emitting Quantum Dot-Based LEDs

**DOI:** 10.3390/nano10122487

**Published:** 2020-12-11

**Authors:** Mariya Zvaigzne, Irina Domanina, Dmitriy Il’gach, Alexander Yakimansky, Igor Nabiev, Pavel Samokhvalov

**Affiliations:** 1 Laboratory of Nano-Bioengineering, National Research Nuclear University MEPhI (Moscow Engineering Physics Institute), 115409 Moscow, Russia; frolova.irina.mephi@yandex.ru (I.D.); igor.nabiev@univ-reims.fr (I.N.); 2 Laboratory of Polymer Nanomaterials and Compositions for Optical Media, Institute of Macromolecular Compounds of the Russian Academy of Sciences, 199004 St. Petersburg, Russia; ilgachdm@hq.macro.ru (D.I.); yak@hq.macro.ru (A.Y.); 3 Laboratoire de Recherche en Nanosciences (LRN-EA4682), Université de Reims Champagne-Ardenne, 51100 Reims, France

**Keywords:** quantum dots, polyfluorene, charge transfer, energy transfer, white-light emission, QDLEDs

## Abstract

Colloidal quantum dots (QDs) are a promising luminescent material for the development of next generation hybrid light-emitting diodes (QDLEDs). In particular, QDs are of great interest in terms of the development of solid-state light sources with an emission spectrum that mimics daylight. In this study, we used CdSe(core)/ZnS/CdS/ZnS(shell) QDs with organic ligands mimicking polyfluorene and its modified derivatives to obtain QD–polymer composites emitting white light. We found that the emission of the composites obtained by spin-coating, being strongly dependent on the chemical structure of the polymer matrix and the QD-to-polymer mass ratio, can be accurately controlled and adjusted to bring its emission spectrum close to the spectrum of daylight (CIE coordinates: 1931 0.307; 0.376). Moreover, the light emission of these composites has been found to be temporally stable, which is due to the minimal structural instability and volume-uniform charge and energy transfer properties. Thus, the use of the synthesized polyfluorene-based organic ligands with controllable chemical structures adaptable to the structure of the polymer matrix can significantly increase the stability of white light emission from QD composites, which can be considered promising electroluminescent materials for fabrication of white QDLEDs.

## 1. Introduction

Colloidal quantum dots (QDs) are luminescent nanocrystals that have great potential for use in many applications. Their unique physical and chemical properties determine a variety of new QD-based trends in biomedicine, laser physics, and optoelectronics [1,2,3,4,5]. The major advantage of QDs is the possibility to control their optical properties, i.e., the luminescence maximum wavelength and absorption spectral range, by varying their physical size. Another important particularity of QD properties is the possibility of obtaining colloidal solutions of QDs that can be used as fluorescent inks, which makes it possible to fabricate functional QD films using relatively cheap methods, such as spin-coating or applying QDs by inkjet printing [6,7,8]. In the field of hybrid optoelectronics, this opens up the opportunity to avoid the use of expensive vacuum deposition technologies, and therefore to simplify, and reduce the cost of, manufacturing microelectronic devices. QDs are of particular interest in the development of solid-state light sources with an emission spectrum that mimics daylight. There are several ways to achieve white light emission using QDs. The first example is a combination of a semiconductor LED emitting in the blue region of the optical spectrum with a layer of red and green QDs deposited on top of it, which yields the desired white light [9]. Another way is to use composites based on QDs and fluorescent organic polymers as an active electroluminescent layer of a hybrid QD-based LED (QDLED) [10,11]. In this case, the combination of the emission spectra of the polymer matrix and embedded QDs mimic the emission spectrum of daylight. The main advantage of this approach is the simplicity and cost-effectiveness of fabricating such LEDs on flexible substrates using the spin-coating or inkjet printing methods, because the emitting layer is deposited from a mixed solution of the polymer and QDs. Another important advantage of this approach is that only two components are used in the composites, which simplifies the process of optimizing the emission spectrum of the fabricated devices, whereas in other options, more than two fluorophores are necessary to obtain white light [9].

However, the incorporation of QDs into a polymer matrix can significantly alter their optical properties [9,12,13], which, as a result, affects the emission spectrum of the composite and, finally, that of the QDLED. Apart from the obvious aggregation-related issues in such systems, various transfer processes can occur between the QDs and the polymer matrix: radiative energy transfer, nonradiative transfer by the FRET mechanism, and charge transfer between the QDs and their environment [13,14,15,16]. For this reason, an important role in the physicochemical stability of QD–emissive polymer composites is played by the QD surface ligands, organic molecules that have a high affinity for both the QD surface and the surrounding media, thus stabilizing the nanocrystals both in a solution and in the solid phase when QDs are embedded in the polymer matrix [9].

In this study, we investigated the effect of polyfluorene and its chemically modified derivatives on the QD photoluminescence (PL) properties in order to design QD–polymer composites with a stable white PL spectrum. We showed that the PL of the composite developed strongly depends on the chemical structure of the polymer matrix and the QD-to-polymer mass ratio. Moreover, we demonstrated that the emission of these composites can be accurately controlled by selecting the polyfluorene derivative with the chemical structure adaptable to the structure of the polymer matrix and adjusted so as to bring this emission spectrum close to the spectrum of daylight.

## 2. Materials and Methods 

**Figure 1:**
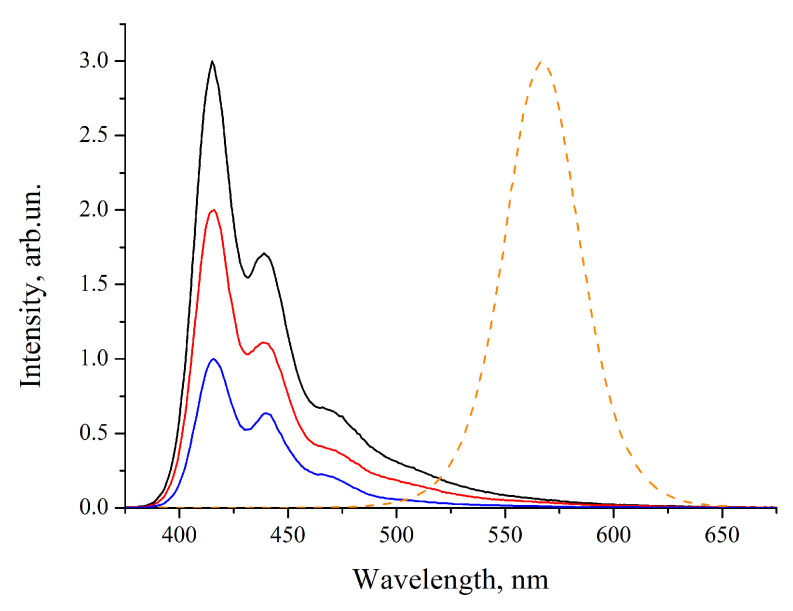
Luminescence spectra of polymer solutions and quantum dots (QDs). Polymer p99 (black), polymer p79 (red), and polymer p133 (blue) in toluene; luminescence spectrum of CdSe/MS QDs in toluene (dashed orange line).

CdSe(core)/ZnS/CdS/ZnS(shell) core/multishell QDs (CdSe/MS) with the luminescence maximum at a wavelength of 569 nm and a full width at half maximum (FWHM) of 40 nm were fabricated by colloidal synthesis according to the technique described by us elsewhere [17]. The synthesis of CdSe cores was carried out by the “hot injection” of trioctylphosphine selenide into a solution of cadmium n-hexadecylphosphonate in 1-octadecene heated to 240 °C. This method allows obtaining QDs with a relatively narrow size distribution of the CdSe cores in the ensemble. A multilayer ZnS/CdS/ZnS shell was deposited on pretreated cores [18] by the method of successive ionic layer adsorption and reaction (SILAR) [17]. The as-prepared QDs were capped with hexadecylammonium palmitate (HDA-PA) ligands; the introduction of these ligands was carried out at the last stage of the QD synthesis to increase their stability during storage and processing. The luminescence spectrum (measured using an Cary Eclipse spectrofluorimeter (Agilent, Santa Clara, CA, USA)) of the QDs used in this study is shown in Figure 1 (dashed orange line). All luminescence spectra reported in this study were recorded under excitation at 350 nm. The luminescence quantum yield (QY) of the as-prepared QDs was found to be 91% using Rhodamine 6G as a reference dye [19], but it dropped to 54% during the purification procedure and sample processing due to charge transfer processes typical of QDs in solution [2,13].

**Figure 2:**
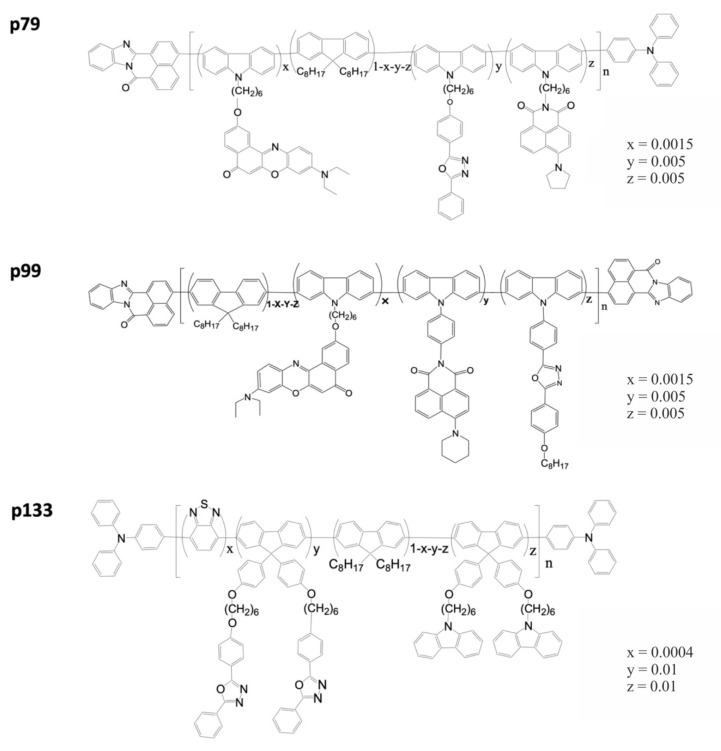
Structural formulas of the p79, p99, and p133 polyfluorene-based polymers.

To study the possibility of using polyfluorene and its chemical derivatives to obtain QD–polymer composites with white light emission for the development of QDLEDs, organic polymers that efficiently fluoresce in the blue region of the optical spectrum were selected. The structural formulas of the polymers are shown in Figure 2. The polymers used were designated p79, p99, and p133. The PL spectra of the polymers are shown in Figure 1 (solid lines, all spectra are normalized to different values for convenience). As seen from these spectra, all the considered polymers have similar optical properties, which are mainly determined by the main chain of the polyfluorene network. All polymers have the first emission maximum at a wavelength of 417 nm and the second maximum at a wavelength of 440 nm, which could be attributed to the exciton–vibronic bands (417 nm 0-0 and 440 nm 0-1) of the long fluorene sequence in the copolymers p99, p79, and p133. The luminescence QYs of the studied polymers in solution were measured using Coumarin 102 as a reference dye and were found to be 74, 82, and 73% for p99, p79, and p133, respectively.

The fabrication of QD–polymer composites was carried out according to the following procedure. First, a portion of solid QDs was weighted and dissolved in toluene, after which the solution was purified twice from excess surface ligands by precipitating QDs with methyl acetate and redissolving them in toluene. After purification, the dry QD residue was redissolved in toluene, and the absorption spectrum of the solution was measured (an Agilent Cary 60 UV-Vis spectrophotometer). The QD concentration was determined using the Beer–Lambert law and the known values of the QD extinction coefficient at the first exciton maximum (100,000 (M × cm)^−1^) and molecular mass of QDs with a PL maximum at 569 nm (200 kDa). The polymers were also dissolved in toluene at a concentration of 10 mg/mL, and then mixed with a QD solution to obtain QD-to-polymer mass ratios of 1:1, 1:10, and 10:1. Then the solutions were applied onto precleaned glass substrates by spin-coating at a spinning rate of 2000 rpm, after which they were dried under normal conditions. As a result of these procedures, thin film samples with different mass ratios between QDs and polymer matrix were obtained. After the film deposition, the luminescence spectra were measured, and then chromatic diagrams were constructed to visualize the characteristics of the radiation chromaticity, and the corresponding CIE 1931 chromaticity coordinates were determined. To study the dynamics of changes in the luminescence of these composites, the photoluminescence spectra of the samples were measured immediately after preparation and after 1, 2, 4, and 7 days of their storage in the dark.

**Figure 3:**
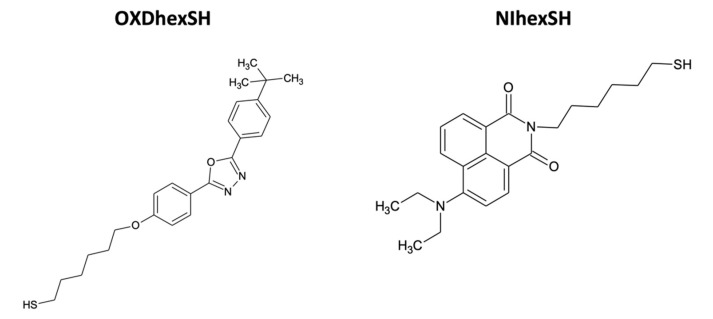
Structural formulas of the OXDhexSH and NIhexSH ligands.

In order to study the effect of QD ligands on the luminescent properties of the composites, the initial CdSe/MS QD ligands were replaced with ligands structurally resembling the polyfluorenes used, NIhexSH, and OXDhexSH (Figure 3). It is assumed that such ligands ensure a more uniform distribution of QDs in the composite. The ligand replacement protocol was as follows. Stock solutions of QDs in toluene were twice purified from excess original ligands as described above. After the second centrifugation, the QD pellet at the bottom of the centrifuge tube was dissolved in toluene in which a 1000-fold excess of the new ligand was previously dissolved. To increase the efficiency of substitution, after the addition of new ligands, the QD solutions were placed in a water bath and kept for 1 h at a 60 °C. Then, the QD solution with the new ligands was precipitated again with methyl acetate and centrifuged, and the procedure for treating QDs with an excess of new ligands was repeated. Finally, two additional sedimentation/redispersion cycles were performed as described above, to obtain pure samples of QDs capped with the NIhexSH or OXDhexSH ligand. These QDs were mixed with different polymer solutions, and thin film samples were prepared by the method described above.

## 3. Results and Discussion

**Figure 4:**
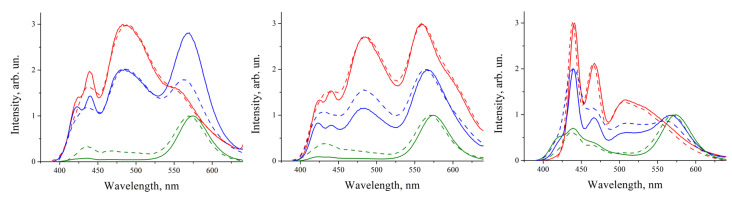
Luminescence spectra of the QD–p79 (**A**), QD–p99 (**B**), and QD–p133 (**C**) composites at various QD-to-polymer concentration ratios, measured immediately after fabrication (solid lines) and after a week of storage under normal conditions (dashed lines). The QD-to-polymer concentration ratios are 1:10 (red), 1:1 (blue), and 10:1 (green).

Figure 4 shows the luminescence spectra of the film composites made from the p79, p99, and p133 polymers and QDs containing the original HDA-PA ligands on their surface. Initially, we compared the luminescence spectra of the solutions of the pristine polymer and QDs with the spectra of their composite form measured on the day of composite film deposition. In the samples with a QD-to-polymer mass ratio of 1:10, the QD luminescence maximum was shifted to shorter wavelengths. In this case, an asymmetric peak was observed at 470–500 nm, in the middle between those of pure polymers and QDs, with a weakly pronounced hump in the long-wavelength region, characteristic of our QDs. As one can see, this effect strongly depended on the structure of the polymer matrix; it was most pronounced for the composites based on the p79 polymer and was almost negligible for the ones based on the p133 polymer. Presumably, this may have been related to the misbalance in the degree of energy or charge transfer from QDs of various sizes in the ensemble to different fragments of polymer chains. However, this hypothesis requires further investigation. For samples with a mass ratio of 1:1, no shift of the QD PL maximum was observed, and for the samples with a mass ratio of 10:1, the QD PL maximum was shifted by 5 nm to longer wavelengths. Apparently, this fact may be explained by the large excess of QDs over the polymer in the prepared samples. Indeed, in the samples with a large content of QDs, the probability of QD aggregation increases, which may be the cause of the red shift of their PL spectrum induced by nonradiative energy transfer (FRET) from smaller to larger QDs [14].

Similar comparison of the luminescence maxima of the polymers showed that, for the composites containing the p79 and p99 polymers at QD-to-polymer mass ratios of 1:1 and 1:10, the first, most intense peak was shifted to longer wavelengths by 7 nm, while the second peak did not change its position. The samples with a QD-to-polymer mass ratio of 10:1 exhibited only one maximum of polymer luminescence, at a wavelength of 430 nm. Regarding the composites based on the p133 polymer, a significant shift to longer wavelengths by 25 nm was observed for both luminescence maxima in the samples with QD-to-polymer ratio mass ratios of 1:1 and 1:10, whereas no PL maximum shift was observed at the QD-to-polymer mass ratio of 10:1.

Analysis of the time course of alteration of the composite film luminescence spectra showed that the main changes in the lineshape of the spectra occurred within the first 4 days after the film deposition. Therefore, we compared the PL spectra of the composites obtained immediately after film fabrication and after a week of their storage in the darkness, assuming that there were no further changes in the spectral characteristics. For all samples based on the p79 and p99 polymers, the luminescence spectrum measured after a week of storage in the dark had a single peak at a wavelength of about 430 nm. In the samples based on the p133 polymer, with QD-to-polymer mass ratios of 1:1 and 1:10, two luminescence peaks of the polymer shifted to the short-wavelength region by 4 nm were observed. The sample with a QD-to-polymer ratio of 10:1 had one emission peak located at 434 nm. These aging effects of the luminescence shift in polymers can be explained by their gradual oxidation by atmospheric oxygen, which has been previously observed [20]. In addition, the spin-coating process allows one to deposit composite films that are thermodynamically metastable, which is followed by phase segregation of the polymer and QDs to a thermodynamically stable two-phase film after a prolonged time, accompanied by spectral modifications of both components. Figure 4 shows that the samples where the amount of polymer either considerably exceeded the mass content of QDs or was approximately equal to it were the most stable over time. It can be assumed that, in the case of a relatively low QD content in the composite, the probability of QD aggregation was also low, which led to a more uniform distribution and better separation of nanoparticles over the volume of the sample. However, the PL spectrum of the obtained composites was strongly shifted towards the blue region.

**Figure 5:**
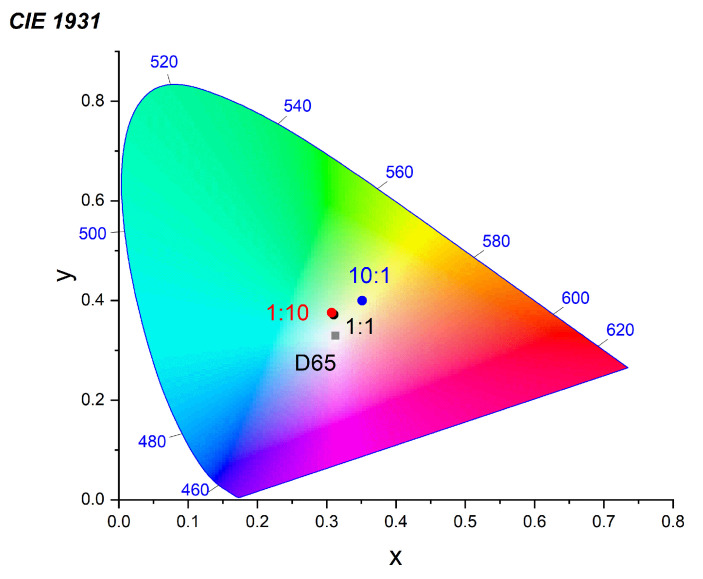
Chromatic diagram of the QD–p99 composite films with different QD-to-polymer mass ratios.

Analysis of the emission color coordinates (Figure 5) of the composite films showed that the QD–p99 composite film with a QD-to-polymer mass ratio of 1:10 (*x* = 0.307, *y* = 0.376) was the closest to the “absolutely white” point (D65) with the coordinates *x* = 0.3128 and *y* = 0.329, which corresponds to the emission of a standard daylight source established by the International Commission on Illumination.

**Figure 6:**
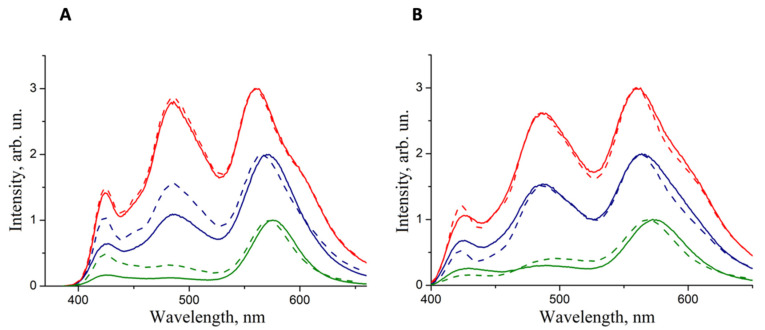
Luminescence spectra of composites consisting of QDs with OXDhexSH ligands and p99 (**A**) and QDs with NIhexSH ligands and p99 (**B**) recorded immediately after composite preparation (solid lines) and after a week of storage under normal conditions (dashed lines). The QD-to-polymer mass ratios in the composites were 1:10 (red lines), 1: 1 (blue lines), and 5: 1 (green lines).

This QD–p99 composite sample also had a greater temporal stability than, for example, the QD–p133 sample with a mass ratio of 10:1, which also had color coordinates close to the coordinates of white light (*x* = 0.320, *y* = 0.368), because the latter exhibited a shift of the luminescence peak and a change in its shape after several days of storage. Thus, the relative drifts of the |Δ*x*| and |Δ*y*| color coordinates for the former sample were, respectively, 5 and 11 times smaller than those of the latter sample. Therefore, further studies on the effect of surface ligands on the luminescence properties of the prepared composites were carried out using polymer p99. In addition, taking into account that the samples with a large excess of QDs were found to be extremely unstable with time, we omitted the highest QD-to-polymer mass ratio of 10:1 in the subsequent experiments in favor of lower contents of QDs in the composite materials. We supposed that the effects of both time-dependent deterioration of QD–polymer composites and large unpredictable spectral drifts of the emission of individual components were caused by a bad compatibility of the original aliphatic ligands of the QDs with the polymer matrix. Indeed, this incompatibility could lead to severe aggregation of QDs in the composite leading to PL quenching and non-uniform energy or charge transfer at the polymer–QD interface. Moreover, this large difference in the chemical structure of the polymer matrix and QD organic ligands could lead to time-dependent reorganization of the components in the bulk composite, leading to phase segregation, which would further alter the composite luminescence properties. Finally, these phase-segregated composites could be supposed to be more prone to polymer oxidation due to the higher permeability of the inhomogeneous composite for atmospheric oxygen and moisture. Therefore, in order to solve these problems, we made an attempt to improve the stability of the luminescence properties of the composite films by using QDs whose surface was better adapted to the structure of the polymer matrix. For this purpose, the composite films based on a mixture of the p99 polymer and QDs coated with the specially synthesized NIhexSH and OXDhexSH ligands were prepared. Figure 6 shows the luminescence spectra of the obtained composites.

Figure 6 shows that the shape of the luminescence spectra of the samples in which the NIhexSH ligand was used were altered significantly less after a week of storage than that of the luminescence spectra of the composites in which QDs with the original aliphatic ligands were used (Figure 4). The positions of the QD luminescence maxima changed by no more than 5 nm (for the sample with a QD-to-polymer mass ratio of 5:1) even after a week of storage, and the polymer luminescence maxima practically did not change their positions. For the composites based on QDs with the OXDhexSH ligands, similar results are observed. However, as can be seen from the spectra, the QD-to-polymer emission intensity ratio changed with time to a greater extent compared to the samples in which NIhexSH was used as a QD surface ligand. In this case, a decrease in the intensity of the QD peaks relative to the polymer peaks was observed. Analysis of the changes in the color coordinates of the radiation (Table 1) showed that the use of the NIhexSH ligand made it possible to significantly increase the stability of the luminescence of the samples under study. These samples remained stable for over a week, whereas the luminescence of the samples with the original ligands degraded as soon as after a day of storage. Indeed, there was no decrease in the intensity of the QD luminescence relative to the polymer, which led to a minor color coordinate drift: by a factor of 1.5 for |Δ*x*| and by a factor of 4 for |Δ*y*|. Furthermore, the luminescence QY of the QD/NIhexSH–p99 film with a QD-to-polymer mass ratio of 5:1, measured in the integration sphere by the method described in [21], was found to be very close to that of the pristine p99 polymer (75% and 82%, respectively). This finding allows us to conclude that even high loading of NIhexSH–capped QDs does not severely affect the luminescence energy efficiency of the polymer matrix.

The data show that the production of QD–polymer composites with a white emission spectrum and temporally stable spectral characteristics requires careful adjustment of the QD-to-polymer mass ratio in the composite, and functionalization of the QD surface with adaptable organic ligands capable of enhancing thermodynamic stability of the nanocrystal dispersion in the matrix and stabilizing the energy- and charge-transfer properties throughout the volume of the composite and in time. We believe that our approach based on the use of polymer-mimicking ligands for preparation of luminescent composites could be extended by using more complex ligand systems that include several types of ligand molecules bearing different structural fragments of the polymer matrix, or by enhancing the affinity of ligand molecules for the QD surface or the native ligand shell and the polymer matrix. The use of the novel QD–polymer composites as the electroluminescent layer of QDLEDs will be the subject of our further research.

## 4. Conclusions

We systematically investigated the optical properties of QD–polymer composites and developed composites based on CdSe(core)/ZnS/CdS/ZnS(shell) QDs and polyfluorene-based ligands emitting white light. Although we found that all of the studied polymers can be used to fabricate QD–polymer composites with a white emission spectrum, their temporal stability and predictability of the spectral coordinates strongly depend on the exact mass ratio between the QDs and polymer matrix and on the type of organic ligands used for the QD stabilization. In order to minimize the phase incompatibility issues caused by the different natures of the original QD ligands and polymer matrix, we successfully used specially synthesized surface ligands with a structure resembling the chemical structure of the polymer matrix to obtain temporally stable composites with white light emission, which could serve as electroluminescent materials for future QDLEDs.

The emission of the composites obtained by spin coating, being strongly dependent on the chemical structure of the polymer matrix and the QD-to-polymer mass ratio, was accurately controlled and adjusted to bring its emission spectrum close to the spectrum of daylight (CIE coordinates: 1931 0.307; 0.376). The data show that the use of the synthesized polyfluorene-based organic ligands with controllable chemical structures adaptable to the structure of the polymer matrix can significantly increase the stability of white light emission from the QD composites, which may be considered promising electroluminescent materials for fabrication of white QDLEDs.

## Figures and Tables

**Table 1:** Emission color coordinates of the QD–p99 films with NIhexSH and OXDhexSH used as the QD surface ligands.

Sample	Color Coordinates					
	Immediately after Preparation		After 7 Days of Storage		*|*Δ*x|*	*|*Δ*y|*
	*x*	*y*	*x*	*y*		
NIhexSH ligand						
1:1	0.334	0.406	0.336	0.428	0.002	0.022
1:10	0.324	0.402	0.322	0.401	0.002	0.001
5:1	0.386	0.423	0.371	0.459	0.015	0.036
OXDhexSH ligand						
1:1	0.356	0.407	0.325	0.385	0.031	0.022
1:10	0.317	0.389	0.312	0.386	0.005	0.003
5:1	0.431	0.440	0.358	0.329	0.073	0.111
White light (D65)	0.3128	0.329				
